# Multi-Fluid Pipeline Leak Detection and Classification Using Savitzky–Golay Scalograms and Lightweight Vision Transformer Featuring Streamlined Self-Attention

**DOI:** 10.3390/s25227001

**Published:** 2025-11-16

**Authors:** Niamat Ullah, Zahoor Ahmad, Jong-Myon Kim

**Affiliations:** 1 Department of Electrical, Electronics and Computer Engineering, University of Ulsan, Ulsan 44610, Republic of Korea; niamat016@mail.ulsan.ac.kr (N.U.); cadet.zahoor@gmail.com (Z.A.); 2 PD Technology Co., Ltd., Ulsan 44610, Republic of Korea

**Keywords:** acoustic emission, Savitzky–Golay scalograms, lightweight vision transformer, artificial neural network

## Abstract

This paper presents a novel pipeline leak diagnosis framework that combines Savitzky–Golay scalograms with a lightweight advanced deep learning architecture. Pipelines are critical for transporting fluids and gases, but leaks can lead to operational disruptions, environmental hazards, and financial losses. Leak events generate acoustic emissions (AE), captured as transient signals by AE sensors; however, these signals are often masked by noise and affected by the transported medium. To overcome this challenge, a fluid-independent detection approach is proposed that begins with acquiring AE data under various operational conditions, including multiple intensities of pinhole leaks and normal states. The transient signals are transformed into detailed scalograms using the Continuous Wavelet Transform (CWT), revealing subtle time–frequency patterns associated with leak events. To enhance these leak-specific features, a targeted Savitzky–Golay (SG) filter is applied, producing refined Savitzky–Golay scalograms (SG scalograms). These SG scalograms are then used to train a Convolutional Neural Network (CNN) and a newly developed lightweight Vision Transformer with streamlined self-attention (LViT-S), which autonomously learn both local and global features. The LViT-S achieves reduced embedding dimensions and fewer Transformer layers, significantly lowering computational cost while maintaining high performance. Extracted local and global features are merged into a unified feature vector, representing diverse visual characteristics learned by each network through their respective loss functions. This comprehensive feature representation is then passed to an Artificial Neural Network (ANN) for final classification, accurately identifying the presence, severity, and absence of leaks. The effectiveness of the proposed method is evaluated under two different pressure conditions, two fluid types (gas and water), and three distinct leak sizes, achieving a high classification accuracy of 98.6%. Additionally, a comparative evaluation against four state-of-the-art methods demonstrates that the proposed framework consistently delivers superior accuracy across diverse operational scenarios.

## 1. Introduction

The Pipelines are an essential infrastructure for transporting vital resources, such as water, oil, and gas, across long distances [1,2,3]. While pipelines are cost-effective and efficient, their prolonged exposure to harsh environmental and operational conditions can lead to structural degradation, resulting in leaks or ruptures. Such leaks have serious implications, ranging from resource wastage and financial losses to environmental contamination and public safety risks [3]. To reduce these risks, early detection of leaks is useful for pipeline operators. Therefore, the development of reliable leak detection systems is important, focusing on both sensitivities to leaks and the ability to minimize false alarms in noisy environments [4,5,6].

Historically, pipeline leak detection has relied on external and internal monitoring methods. External methods, such as visual inspection and ground-penetrating radars, often provide limited coverage and are generally suitable only for surface-level inspections. Internal methods, however, have proven more effective for real-time monitoring, allowing for the detection of leaks as they occur [6]. Negative pressure wave (NPW) analysis, accelerometer-based systems, time-domain reflectometry, and distributed temperature sensing have all been used to monitor pipeline health [7]. For example, NPW techniques can detect pressure drops when a leak occurs but are often limited by their sensitivity to small leaks and susceptibility to operational noise. Likewise, accelerometer-based monitoring can capture changes in pipeline vibrations due to structural abnormalities, though it may produce false positives due to background mechanical noise [8].

Among these techniques, AE technology stands out for its sensitivity and ability to provide real-time monitoring of transient events associated with pipeline faults [9]. AE technology detects stress waves emitted by structural changes, such as leaks or cracks, in pipelines. These waves create transient signals, known as AE events or AE hits, which can be analyzed to assess the pipeline’s condition. AE technology has been widely adopted in other fields, such as structural health monitoring of bridges, aircraft, and industrial machinery, due to its high sensitivity and rapid response [10]. However, the application of AE to pipeline monitoring presents unique challenges, particularly regarding the extraction of relevant features from noisy AE signals, which are often affected by background interference from equipment or environmental factors [11].

To overcome these challenges, researchers have explored various feature extraction methods for analyzing AE signals. Time-domain features, including peak amplitude, rise time, and counts, are straightforward to compute and capture transient AE events; however, these features are often sensitive to noise and may generate false alarms [12]. Frequency-domain features offer insight into the spectral content of AE signals and are useful for detecting repetitive patterns; however, they can struggle with non-stationary signals, which are common in pipeline monitoring. Consequently, time-frequency domain techniques, such as wavelet transform (WT), have been widely employed to capture both temporal and spectral information simultaneously, enhancing the robustness of leak detection in pipelines [13].

Wavelet-based methods have demonstrated considerable success in detecting pipeline leaks [14]. For instance, Xiao et al. [15] used wavelet-based feature extraction combined with support vector machines (SVMs) to classify leak and non-leak states, achieving a significant increase in detection accuracy [16]. Similarly, empirical mode decomposition (EMD) [17] and its variations, such as variational mode decomposition (VMD) [18] and local mean decomposition (LMD) [19], have been applied to decompose non-stationary AE signals into intrinsic modes, which capture oscillatory behaviors associated with leaks. These decompositions have been effective in identifying minute leak signatures, but they rely on selecting optimal basis functions or modes, which may not generalize well across different pipeline environments. Additionally, time-frequency techniques like WT and EMD are computationally intensive, which can hinder their application in large-scale or real-time monitoring systems [20].

Machine learning (ML) has emerged as a powerful tool for pipeline leak detection, particularly due to its ability to automatically recognize patterns in large datasets. Traditional ML approaches, such as SVM, k-nearest neighbors (KNN), and decision trees, have been employed to classify pipeline conditions based on features extracted from AE data. Banjara et al. [21] used SVM and relevance vector machines (RVMs) to classify pipeline leak states based on waveform indicators from AE signals, achieving reliable detection under controlled conditions. Furthermore, hybrid models combining ML and statistical methods have shown promise in pipeline health monitoring. Rai et al. (2021) [22], for example, proposed a pipeline health index that integrates multiscale analysis with the Kolmogorov–Smirnov (KS) test and Gaussian mixture models to quantify leak severity, creating a comprehensive health assessment model for pipelines.

Despite the advancements made with traditional ML methods, their application in pipeline leak detection has limitations. These models often require extensive training data, which can be challenging to obtain in industrial settings where leaked data are scarce. Moreover, traditional ML models may struggle with the high dimensionality of AE data [1,23], which often includes both local and global information relevant to leak patterns. To address these limitations, recent studies have increasingly turned to deep learning (DL), specifically CNNs, which excel at capturing spatial features in data and are particularly effective with image-like inputs. The application of CNNs in pipeline leak detection has shown promising results, especially with the use of CWT to convert AE signals into scalograms, visual representations of time-frequency distributions [24]. CNNs can analyze these scalograms, automatically learning both local and global features indicative of leaks. For instance, Ahmad et al. [25] demonstrated that CNNs trained on scalograms could accurately classify leak and non-leak conditions, showing considerable robustness to noise. However, CWT-based methods can face challenges, particularly in balancing time and frequency resolution and managing the high computational cost associated with processing large scalograms. To address these issues, some studies have explored combining CWT with other feature extraction and processing techniques to create a more comprehensive representation of AE signals. For example, Sajjad et al. [26] enhanced leak detection by processing CWT scalograms with convolutional autoencoders (CAEs), which reduced the data’s dimensionality while retaining important leak-related features. Such hybrid approaches highlight the potential of combining multiple analysis techniques to enhance leak detection performance [27]. Yang et al. [28] show that pairing a digital twin with real data and a Bayesian network can untangle composite faults in subsea control systems, using a simple Bernoulli-based physical model and a quick cross-validation pass for single faults, with strong results on South China Sea field data. In a companion study, enhance the twin with cross-validation and feedback to curb model–plant mismatch, improving diagnosis of minor faults over multi-day operations [29]. However, computational efficiency remains a challenge, especially in large-scale or real-time applications where quick response times are essential [30].

To overcome the challenges of industrial noise, high computational costs, and the risk of missing local or global features, we propose a novel hybrid lightweight model for pipeline leak detection. Our approach employs a SG filter to smooth scalograms, effectively reducing noise while preserving critical leak signatures, and integrates ResNet18 for local feature extraction, a Lightweight ViT with reduced embedding dimensions and fewer layers for global context, a streamlined self-attention mechanism for refined feature interactions, and an ANN for classifying the conditions: “Leak size 0.5 mm,” “Leak size 1 mm,” and “Normal”.

The primary contributions of this study are as follows:

1. We introduce a SG-scalogram preprocessing for AE signals that suppresses industrial noise while preserving sharp leak transients (window = 11, order = 2), thereby enhancing signal quality for precise leak identification and yielding accuracy gains of +20.3% and +13.9% over time- and frequency-domain baselines, respectively.
2. A LViT-S is introduced, featuring reduced embedding dimensions, fewer Transformer layers, and a streamlined self-attention mechanism, optimizing computational efficiency while preserving reliable feature extraction for real-time applications.
3. The proposed work utilizes a triple-stream feature fusion approach, integrating ResNet18’s localized spatial features with Lightweight ViT’s global contextual features. Self-attention refines relational features, markedly enhancing leak-specific anomaly detection.
4. Industrial fluid pipeline data is used in this study for leak detection and size identification using end-to-end deep learning models.

The rest of this study is structured as follows. Section 2, laying out the essential theoretical foundations. Section 3 introduces the proposed methodology, elaborating on the approach. Section 4 examines and analyzes the findings. Lastly, Section 5 wraps up the study, highlighting the main results and their implications.

## 2. Fundamental Concept

### 2.1. CWT

**Figure 1:**
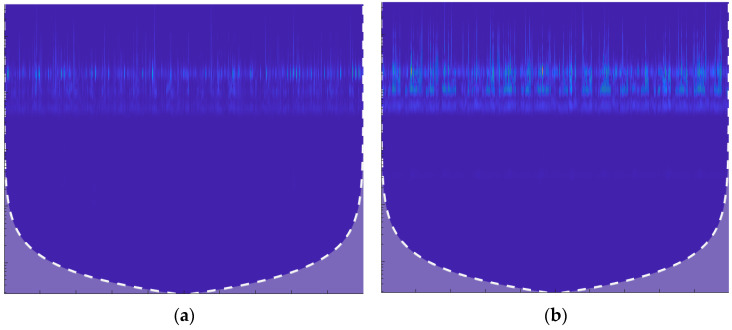
Scalograms of AE Signals Using Continuous Wavelet Transform (CWT). (**a**) Scalogram of the leak signal (1.0 mm pinhole at 13 bar pressure), exhibiting high-energy bursts at specific frequencies. (**b**) Scalogram of the normal signal (13 bar pressure), showing a more consistent and uniform frequency distribution over time, indicative of stable pipeline operation.

In this study, CWT processes raw AE data from R15I-AST sensors positioned at 2500 mm and 0 mm along a 304 stainless steel pipeline, transforming the time-series signals x(t) into 2D scalograms. We use CWT because it excels at capturing transient, non-stationary events like leaks in noisy industrial AE data, offering superior time-frequency resolution over traditional methods. The CWT is defined as:

(1) $$W_{x}\left(s,\tau \right)=\frac{1}{\sqrt{s}}\int_{-\infty }^{\infty }x(t)\;.\;\psi^{*}\left(\frac{t-\tau }{s}\right)dt$$

where ψ(t) is the mother wavelet, scaled by s and shifted by τ with ψ∗ as its conjugate, yielding a scalogram ∣Wx(s,τ)∣2∣. CWT detects subtle machinery leak anomalies unmissable in noise. Figure 1 below illustrates the 2d scalograms of leak and normal signals.

### 2.2. Savitzky–Golay Filter

The SG filter smooths data by fitting a polynomial within a sliding window to reduce noise while keeping important features, like peaks and edges, intact. For each point in the data, the filter looks at a small window of surrounding points, fits a polynomial to them, and replaces the central point with the polynomial’s result. This approach smooths the data while preserving sharp transitions.

(2) $$y_{i}^{\prime }=\sum_{j=-m}^{m}c_{j}\;.\;y_{i+j}$$

where $y_{i}^{\prime }$ is the new, smoothed value, $y_{i+j}$ are the original points in the window, $c_{j}$ are coefficients based on the polynomial fit, and m is half the window size.

**Figure 2:**
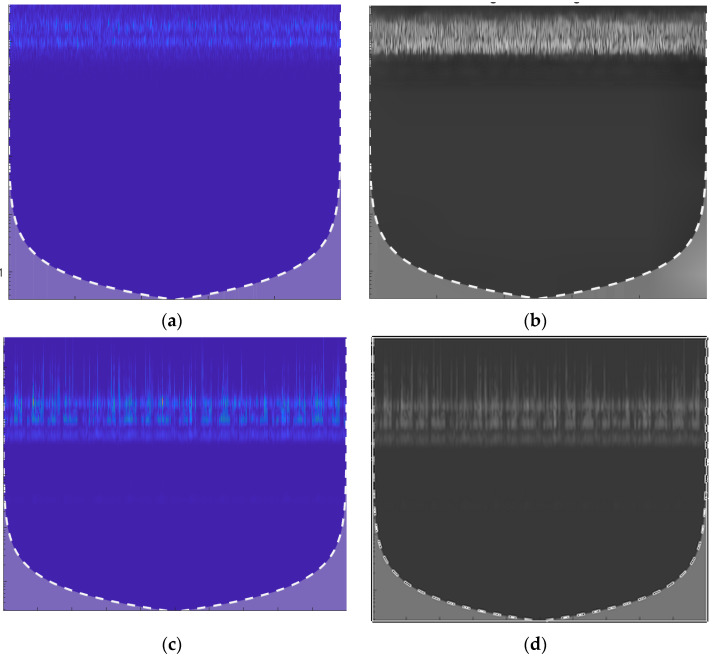
A CWT Scalogram. (**a**) Scalogram of a leak signal (1.0 mm pinhole at 13 bar pressure. (**b**). The same leak signal after SG filtering, smoother and clearer, with noise reduced, but the leak’s key features still stand out. (**c**) Scalogram of a normal signal. (**d**) The normal signal’s scalogram after SG filtering appears even cleaner, with background noise minimized, highlighting the stable operation of the pipeline.

Figure 2 shows the applied filter image with a window size of 11 (covering 5 points on each side) and a polynomial order of 2 (quadratic). This setup reduces noise but keeps the details clear, making it perfect for applications where smooth data is required without losing key features.

### 2.3. RESNET-18

**Figure 3:**
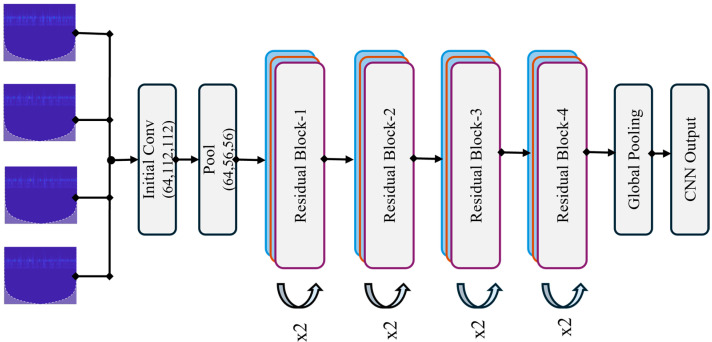
Architecture diagram of ResNet-18 for extracting local spatial features in the proposed Hybrid Lightweight Model for Pipeline Leak Detection. The four input images at the top represent sample SG-refined scalograms from the four experimental datasets (Dataset 1: Water, 13 bar, 1.0 mm leak; Dataset 2: Water, 18 bar, 0.5 mm leak; Dataset 3: Gas, 13 bar, 1.0 mm leak; Dataset 4: Gas, 18 bar, 0.3 mm leak), demonstrating the diversity of time-frequency patterns processed by the network.

CNN is widely used in image analysis due to its ability to extract spatial patterns through hierarchical feature extraction. CNN consists of multiple layers, where each convolutional layer applies a set of filters to learn specific patterns within localized regions of an image. In the context of AE-based scalogram analysis, CNN helps identify localized leak-related features that may be displayed as high-energy bursts or transient anomalies within the time-frequency domain representation of AE signals. The core of CNN architecture is the convolution operation, which extracts features by applying filters across the input data. Mathematically, the convolution operation between an input image I and a filter (or kernel) K can be expressed as:

(3) $$\left(I*K\right)\left(x,y\right)=\;\sum_{i=-m}^{m}\sum_{j=-n}^{n}K\left(i,j\right).I(x+i,\;y+j)$$

where $\left(x,y\right)$ denotes the position in the feature map, m and n are the filter dimensions, I(x+i, y+j) represents the pixel values in the local region, and $K\left(i,j\right)$ is the kernel weight at the position $\left(i,j\right)$. This operation produces a feature map F, which highlights the patterns detected by the filter. By stacking multiple convolutional layers, the CNN learns increasingly complex features, from simple edges to more abstract representations, enabling it to extract detailed local structures within the scalogram images. The architecture diagram of RESNET 18 is shown in Figure 3.

In deeper CNN architectures, such as ResNet-18, residual connections are introduced to combat the vanishing gradient problem and improve training stability. Residual learning is achieved by introducing shortcut connections, where the output of a layer is combined with its input through an addition operation:

(4) $$y=F\left(Χ,\;\left\{W_{i}\right\}\right)+Χ$$

where y is the output of the residual block, $F\left(Χ,\;\left\{W_{i}\right\}\right)$ represents the learned residual function with weights $W_{i}$, Χ is the input to the residual block. The residual connection allows the network to learn identity mappings more easily, enhancing gradient flow and allowing for the construction of deeper networks. In this study, ResNet-18 is used to extract local features from the scalograms, using its capacity for learning both shallow and deep features that are useful for identifying leak-specific patterns within the AE scalograms.

### 2.4. Lightweight Vision Transformer

**Figure 4:**
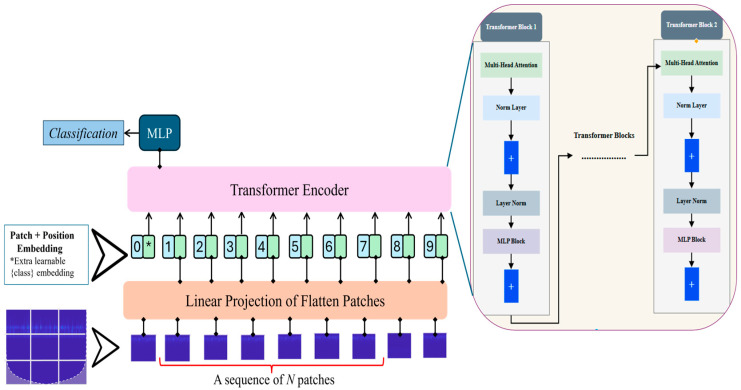
Architecture diagram of Vision Transformer for extracting global features in the proposed Hybrid Lightweight Model for Pipeline Leak Detection. ‘*’ simply marks the class token, this token is the one the model uses to make the final classification.

Transformers have revolutionized sequence modeling and are increasingly applied to image analysis through ViTs. Unlike CNN, which extracts local patterns through convolution, ViTs use self-attention mechanisms to capture both local and global dependencies across an entire image. This capability is particularly useful for AE-based leak detection, where the global scalogram context can indicate diffuse or complex leak signatures. In ViTs, the input image is divided into non-overlapping patches, each of size P ×P. These patches are flattened into vectors and linearly projected into a D-dimensional embedding space:

(5) $$z_{0}^{p}=Flatten\left(x_{i,j}^{p}\right)W_{E}+\;E_{pos}$$

where $z_{0}^{p}$ represents the embedding of the p−th patch, $x_{i,j}^{p}$ pixel in the p−th patch, $W_{E}$ is the linear embedding matrix, and $E_{pos}$ is the positional encoding added to maintain spatial information. The self-attention mechanism in transformers calculates attention scores across the entire set of patches, allowing the model to focus on relevant parts of the image. For each patch, self-attention computes a weighted sum of all other patches based on similarity scores. Given a set of input embeddings $z_{0}^{p}$ the self-attention operation computes queries Q, keys K, and values V as follows:

(6) $$Q=ZW_{Q},\;\;K=ZW_{K},\;\;V=ZW_{V}$$

where $W_{Q}$, $W_{K}$ and $W_{V}$ are learnable weight matrices. The self-attention mechanism enables the model to extract relationships across distant parts of the image, making it effective for identifying global patterns indicative of leaks. In this study, we employ the ViT-B16 model to extract global features from scalograms, complementing the localized feature extraction performed by CNN. This combined approach allows the model to extract both fine-grained and broad contextual information essential for accurate leak detection. Figure 4 illustrates the architecture diagram of vision transformers.

### 2.5. Multi-Scale Feature Fusion with Lightweight RESNET-ViT for Pipeline Leak Detection

In pipeline leak detection using AE scalograms, local features refer to fine-scale patterns such as sharp bursts or textural irregularities that signify the immediate presence of small leaks, while global features encompass broader time frequency signal variations that reveal distributed or complex leaks affecting the overall system; combining both types of features, as enabled by CNNs and vision transformers, provides enhanced sensitivity and robustness under industrial conditions [31].

The process starts with raw AE data collected from industrial pipelines under various conditions (different pinhole sizes and normal operation). This data is transformed into scalograms $X{\in R}^{N\times H\times W\times 3}$ using the CWT, which converts transient signals into 2D time-frequency representations. The SG filter smooths these scalograms, preserving critical leak signatures while reducing noise.

Each scalogram is resized from its original dimensions to 224 × 224 pixels using interpolation (Equation (7)), ensuring uniformity for the neural networks:

(7) $$X^{\prime }=Resize(X),\;X\in R^{N\times 224\times 224\times 3}$$

The resized scalograms are converted into PyTorch tensors, reordering dimensions from N×H×W×C to N×C×H×W and scaling pixel values from (Equation (8)) [0, 255] to [0, 1].

(8) $$X_{temp}^{\prime \prime }=toTensor(X^{\prime }),\;X_{temp}^{\prime \prime }\in R^{N\times 224\times 224\times 3}$$

These tensors are then normalized using the ImageNet mean (μ) and standard deviation (σ) statistics (Equation (9)). This sequence of preprocessing converts variable-sized scalograms into normalized, uniform RGB tensors suitable for input into ResNet18 and the Lightweight ViT models.

(9) $$X_{i,c}^{\prime \prime }=\frac{X_{temp,i,c}^{\prime \prime }-\mu_{c}}{\sigma_{c}},\;c\in \{0,1,2\}$$

The preprocessed X′′ is fed into a pre-trained ResNet18, designed to capture local spatial features such as edges, textures, and patterns in the SG scalograms. The final fully connected layer is replaced with an identity function, preserving the learned features (Equation (10)).

(10) $$F_{CNN}=Resnet(X^{\prime \prime };\theta_{CNN}),\;F_{CNN}\in R^{N\times 512}$$

In parallel, X′′ the same input is processed by a Lightweight Vi optimized for real-time performance and reduced complexity. The normalized scalogram is divided into 196 non-overlapping patches (16 × 16 pixels each). Each patch (flattened to 768 dimensions) is linearly projected to a 256-dimensional space (Equations (11)–(13)), followed by the addition of positional embeddings $P_{pos}$ and the prepending of a [CLS] token, which aggregates the global representation of the input (Equations (14) and (15)).

(11) $$E^{\prime \prime }=X^{\prime \prime }W_{E}+b_{E}$$

(12) $$W_{E}\in R^{768\times 256}$$

(13) $$E\in R^{N\times 196\times 256}$$

(14) $$E^{\prime \prime }=[\left[CLS\right],E+P_{pos}]$$

(15) $$E^{\prime \prime }\in R^{N\times 197\times 256}$$

Next, the embedded patches pass through four transformer encoder layers, each with four-head self-attention (Equations (16)–(19)) and a feed-forward network (Equation (20)). The final output from the [CLS] token after the fourth layer (Equation (21)) encapsulates the global contextual features present in the SG-scalogram.

(16) $$Q,K,V=E^{\prime \prime }W_{Q,K,V}$$

(17) $$Q,K,V\in R^{N\times 197\times 64}$$

(18) $$A=softmax(\frac{QK^{T}}{\sqrt{64}}),\;{head}_{i}=AV$$

(19) $$MHSA=concat({head}_{1},{head}_{2}\ldots {head}_{4})W_{o}$$

(20) $$FFX\left(x\right)=W_{2}\;.\;GELU\left(W_{1}x+b_{1}\right)+b_{2}$$

(21) $$F_{ViT}=E_{\left[CLS\right],4}^{\prime \prime },\;F_{ViT}\in R^{N\times 256}$$

To unify both representations and reduce dimensionality, the outputs from CNN and ViT branches are projected into a 128-dimensional latent space (Equations (22) and (23)). A streamlined self-attention block is then applied to refine the interdependencies between local and global features, thereby enhancing the signal relationships applicable to leak characterization (Equations (24)–(26)).

(22) $$F_{combined}^{\prime }=F_{combined}W_{proj}$$

(23) $$W_{proj}\in R^{768\times 128}$$

(24) $$Q=F_{combined}^{\prime }W_{K},\;V=F_{combined}^{\prime }W_{V}$$

(25) $$W_{Q},W_{K},W_{V}\in R^{N\times 256}$$

(26) $$F_{SA}=AV,\;F_{SA}\in R^{N\times 64}$$

To intuitively understand the function of the streamlined self-attention mechanism in our fused feature representation, think of it as a vigilant operator in a busy industrial control room, where AE signals from pipelines compete with operational chatter similar to fluid turbulence or pump vibrations that can obscure subtle leak-induced transients. The CNN branch, specifically ResNet-18, initially detects these local “alerts,” such as sharp edges or high-energy bursts in the SG-refined scalograms. Meanwhile, the Lightweight ViT captures the broader “context,” identifying recurring time-frequency patterns across the entire signal that may suggest systemic anomalies under various pressures (13 or 18 bar).

**Figure 5:**
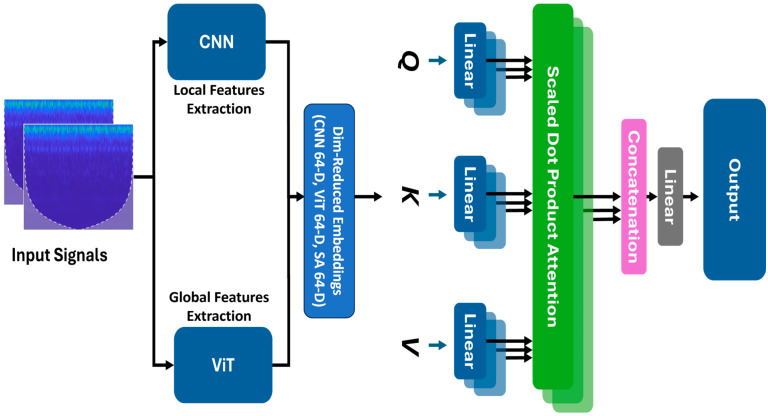
Self-attention fusion in the proposed hybrid CNN–ViT pipeline. SG-scalogram inputs are encoded in parallel by ResNet-18 (local features) and a lightweight ViT (global features). Dimension-reduced embeddings are linearly projected to Q, K, and V, then fused using scaled dot-product attention; the attended features are concatenated and passed through a linear head for classification.

Simply concatenating these 512-D and 256-D features risks obscuring leak signatures among the noise-dominated elements. However, self-attention acts as a dynamic referee: by computing adaptive weights through scaled dot-product attention (Equations (24)–(26)), it evaluates how the local transients detected by the CNN align with the global relationships identified by the ViT. This process amplifies correlated leak harmonics, such as those associated with leaks in gas or water flows, while reducing uncorrelated noise like consistent low-amplitude vibrations in normal conditions. The detailed shown in Figure 5.

The result is a more cohesive 64-D vector (F_SA) that integrates with the projected outputs from both the CNN and ViT to create a robust 192-D feature set for ANN classification. This approach is supported by the model’s impressive accuracy of 98.6% and t-SNE visualizations (Figure 11), which demonstrate a clear separation of leak classes from noise. Essentially, self-attention does more than just merge features; it “listens” selectively, mimicking human intuition to isolate genuine threats in noisy environments, thereby enhancing the framework’s reliability for real-world pipeline monitoring. Next, each feature stream undergoes individual projection (Equations (27)–(29)), and the resulting vectors are concatenated into a single compact representation (Equation (30)). Equation (31) defines the final fused feature vector, which integrates boundary-level leak anomalies (local), distribution-level context (global), and refined inter-feature relationships (attention).

(27) $$F_{CNN}^{\prime }=F_{CNN}W_{proj1}$$

(28) $$F_{ViT}^{\prime }=F_{ViT}W_{proj2}$$

(29) $$W_{proj1}\in R^{512\times 64}$$

(30) $$W_{proj2}\in R^{256\times 64}$$

(31) $$F_{final}=[F_{CNN}^{\prime },F_{ViT}^{\prime },F_{SA}]\in R^{N\times 192}$$

This unified feature vector is passed to a two-layer ANN classifier (Equations (32) and (33)), which outputs logits for three classes: 0.5 mm leak, 1 mm leak, and normal condition. Class probabilities are computed using a SoftMax function (Equation (34)), and the model is trained using cross-entropy loss (Equation (35)). The network is optimized using the Adam optimizer $\eta =10^{-4}$, while the base encoder weights (for ResNet18 and ViT patch embedding layers) remain fixed. Table 1 summarizes the contribution of each architectural component, demonstrating how localized CNN features, global ViT representations, and streamlined attention modules interact to produce a robust, lightweight model suitable for real-time, multi-scenario pipeline monitoring.

(32) $$Z_{1}=W_{1}F_{final}+b_{1},\;W_{1}\in R^{128\times 192},\;H_{1}=ReLU(Z_{1})$$

(33) $$Z_{2}=W_{2}H_{1}+b_{2},\;W_{2}\in R^{3\times 128}$$

(34) $$P\left(y_{i}=k\right)=\frac{exp(Z_{2,i,k})}{\sum_{j}exp(Z_{2,i,k})}$$

(35) $$L=-\frac{1}{N}\sum_{i=1}^{N}\sum_{k=0}^{2}y_{i,k}log(P\left(y_{i,k}\right))$$

## 3. Proposed Method

**Figure 6:**
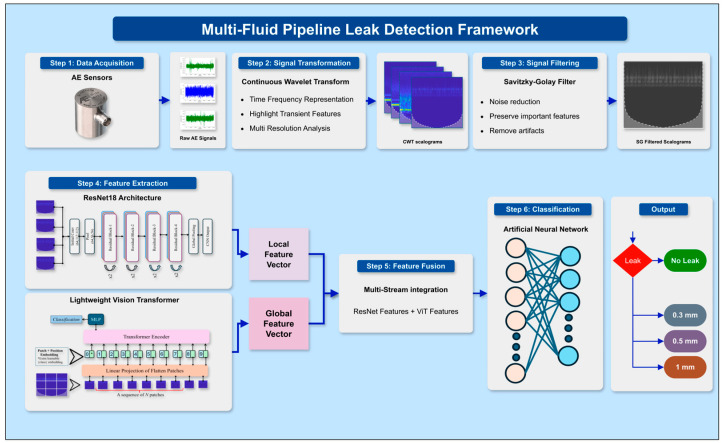
Pipeline leak detection framework of the proposed Hybrid Lightweight model, highlighting feature Extraction with ResNet18 and Lightweight ViT. ‘*’ simply marks the class token, this token is the one the model uses to make the final classification.

Figure 6 shows the detailed architecture diagram of the proposed model, and a step-by-step explanation is given below.

**Step 1:** AE time series data from pipeline systems under both leak and normal operating conditions. To simulate real-world leak scenarios, different pinhole sizes are introduced to create a variety of leak intensities. This helps in developing a robust dataset that represents diverse operational states.

**Step 2**: The acquired AE time series data is transformed into 2D representations using CWT. These CWTs provide an insightful view of how the frequency components of the signal vary over time, making it easier to distinguish between normal and leak conditions based on their unique time-frequency characteristics.

**Step 3:** To enhance the quality of these scalograms, an SG filter is applied. This step is important for noise reduction; it removes unwanted fluctuations while preserving the critical features of the signals. By retaining essential time-frequency information, the filtered scalograms are clearer and more representative of the real AE activity.

**Step 4:** Feature Extraction via CNN and ViT: The refined scalograms are then fed into a combination of deep learning models for feature extraction: CNN Extracts spatial features such as textures and edges, which are highly useful in distinguishing between leak and normal patterns. A streamlined Lightweight Vision Transformer is introduced with smaller embedding sizes, fewer layers, and an enhanced self-attention process, delivering strong feature extraction for real-time use in low-resource industries.

**Step 5:** Then, it employs an advanced three-way feature fusion, integrating ResNet18’s detailed spatial features, the Lightweight ViT’s broad contextual insights, and refined relational features from a simplified self-attention layer. This greatly enhances leak anomaly detection to create a strong feature vector.

**Step 6:** The concatenated feature vector is passed into an ANN for classification. The ANN uses these features to determine whether the observed condition represents a leak or a normal operating state. This step forms the core decision-making part of the pipeline.

## 4. Results and Discussion

This section provides an in-depth explanation of the experimental setup and results.

### 4.1. Experimental Setup for Pipeline Testing

**Figure 7:**
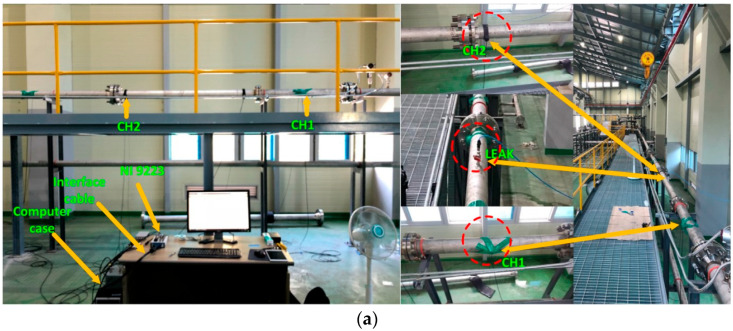

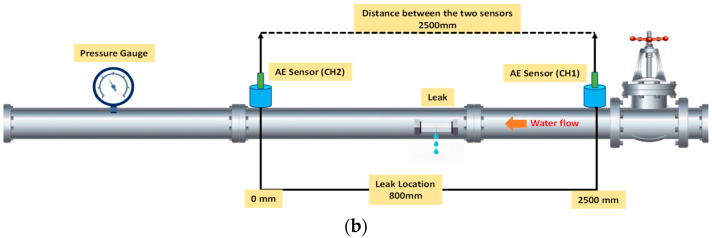
Test setup for the pipeline under test. (**a**) Pictorial view, (**b**) Schematic diagram of the AE sensors data acquisition system.

To collect AE sensor data, a segment of a large industrial fluid pipeline was utilized. Figure 7a shows the layout of the pipeline test configuration, with Figure 7b illustrating detailed schematics of the testbed. Key parameters set for the experiment include the locations of three AE sensors positioned at 0 mm, 1600 mm, and 2500 mm along the pipeline. The sensors have peak sensitivities of 109 dB and 22 dB, depending on the reference parameter, and they operate within a frequency range of 50 to 400 kHz, with resonant frequencies at 75 kHz and 150 kHz. The directionality of the sensors is ±1.5 dB, and they are functional across temperatures from 35 °C to 75 °C. The pipeline is made of stainless steel 304, with a thickness of 6.02 mm and an outer diameter of 114.3 mm.

To simulate leaks of different magnitudes, an electric drill was used to create a hole in the pipeline, and a fluid control valve was welded near the drilled hole. Water was circulated through the pipeline as the fluid medium for all tests.

#### 4.1.1. Development of Acoustic Emission Data Acquisition System

The AE data acquisition process, shown in Figure 6, involved a structured setup using high-sensitivity R15I-AST sensors from MITRAS Corporation. The system was powered by a National Instruments (NI) NI-9223 module, which features a 16-bit analog-to-digital converter (ADC) with adjustable sampling rates and connects via a universal standard bus interface. The recorded AE data was stored on a computer with a one-terabyte capacity, fully compatible with the NI-9223 module.

To monitor the fluid pipeline, R15I-AST AE sensors were attached using plastic tape for secure positioning and a specialized gel to ensure optimal sensor contact with the pipeline surface. Data acquisition was managed through custom software developed by the Ulsan Industrial Artificial Intelligence Laboratory, which integrated NI interface libraries with Python to control the data recording process. Prior to leak data collection, the AE sensors were calibrated and tested for sensitivity using the Hsu–Nelson method. Once the system’s functionality and sensor accuracy were verified, a high-quality dataset of pipeline leak signals was obtained. Table 2 contains the detailed configuration and specification of R15I-AST AE Sensor.

#### 4.1.2. Dataset Collection and Description

This study gathered AE datasets from a pipeline operating under normal and leaking conditions utilizing an AE sensor. The normal state was defined by keeping the control valve closed, while a centrifugal pump (CP) set to either 13 or 18 bars maintained the pipeline fluid pressure. The temperature during data collection was around 25 °C. For each pressure setting, data were recorded at a 1 MHz sampling frequency. AE signals were captured for each pressure condition across distinct leak sizes.

The data collection procedure began by closing the fluid control valve and turning on the CP to maintain pipeline pressures at 13 bar and 18 bar. Water was transported through the pipeline at both pressures for two minutes each, contributing to dataset-1 and dataset-2 as a normal pipeline condition. After completing data collection for the normal state, the control valve was opened to simulate leaks opening, with data recorded continuously for another four minutes under both pressures separately contributing to dataset-1 and dataset-2 as leak samples. This procedure was repeated for gas transportation at both pressure levels and at different leak sizes. The samples obtained from gas are named dataset-3 and dataset-4. A summary of the collected datasets is shown in Table 3.

#### 4.1.3. Experimental Setup

The simulation experiments were run on a system equipped with a 7th Generation Intel^®^ Core™ i7-7700K processor (3.60 GHz, 4 cores/8 threads), 16 GB of RAM, and an NVIDIA^®^ GeForce Ti GPU (11 GB GDDR5X memory, 3584 CUDA cores). All algorithms were created and executed using Python 3.10.11, with Keras and TensorFlow 2.12.0 as the main deep learning frameworks, and Pandas 1.5.3 for efficient data handling and analysis.

**Figure 8:**
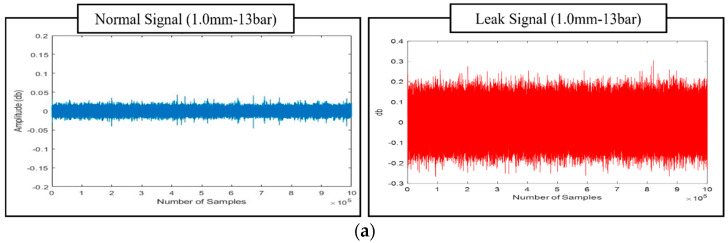

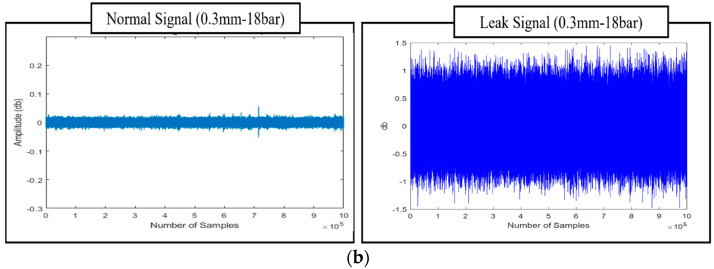
Detailed Comparison of AE sensor Signals for Leak and Non-Leak Scenarios, Illustrated Across Two Pressure Levels: (**a**) Signals Captured at 13 Bar Pressure, Highlighting Distinctive Patterns; and (**b**) Signals Recorded at 18 Bar Pressure, Showcasing Variations in AE Characteristics Under Different Operational Conditions.

Figure 8 provides a detailed comparison of AE signals across different conditions. Figure 8a shows AE signals for leak and non-leak scenarios at 13 bars, while Figure 8b demonstrates the signal variations between leak and non-leak states at 18 bar, highlighting how AE signals differ under various pressures.

### 4.2. Surveillance Zone Identification

The assessment of the surveillance zone, as per ISO standard 18211:2016, involves evaluating the attenuation of AE signals from the noise generated by the AE source. This assessment is conducted before data is collected from the AE sensor. In audio engineering, the term attenuation refers to the reduction in signal intensity, which is usually measured in decibels (dB). The attenuation profile of an AE sensor can be established by applying the equation below.

(36) $$A_{dB}=20Log\frac{V}{V*}$$

Equation (36) represents the measured voltage (V) and the reference voltage (V∗). The phrase “measured AE potential” refers to the AE signal obtained from the AE sensor. In the context of AE, a reference level of 0 dB indicates an AE signal potential of 1 µV at the AE sensor when no amplification is applied.

**Figure 9:**
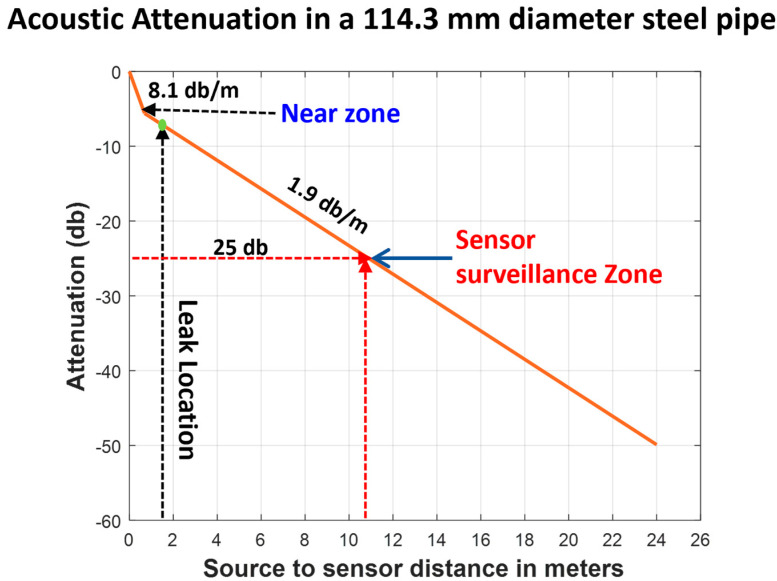
Acoustic emission signal attenuation characteristics in 304 stainless steel pipeline (114.3 mm outer diameter).

The current research utilizes the HSU-Nielsen test as a source of AE to evaluate the attenuation properties of the AE sensor. The HSU-Nielsen test involves conducting a pencil-led break test, where a lead with a diameter of 0.5 mm is placed on the surface of the pipeline to trigger an acoustic emission event. The AE hits recorded in the HSU-Nielsen test display characteristics similar to AE sources typically linked to natural events, like leaks. Figure 9 depicts the attenuation properties of a fluid-filled industrial pipeline with an outer diameter of 114.3 mm. To ensure optimal performance of the R15I-AST AE sensor on the pipeline, the distance between the two sensors should be within a specified range to maintain attenuation below 25 dB. According to the information shown in Figure 9, it is evident that the defined level of attenuation is noted at a distance of 10 m. By placing an AE sensor R15I-AST on an industrial pipeline that has an outer diameter of 114.3 mm, it is possible to attain effective monitoring coverage up to a distance of 10 m. When the attenuation of the acoustic signal goes beyond 25 dB, the AE becomes apparent, registering at levels under 10% of the maximum peak value of the AE signal. As a result, differentiating between the noise produced by leaks and the surrounding noise presents a difficult challenge. This study proposes using an adaptive threshold of 10% of the peak value to distinguish acoustic emission (AE) events from background noise. This approach allows for effective leak detection at a distance of 10 m (10,000 mm) using a single R15I-AST AE sensor.

### 4.3. Evaluation Metrics

To thoroughly assess the effectiveness of our proposed hybrid lightweight model, combining ResNet18, a Lightweight Vision Transformer, and streamlined self-attention, four key performance metrics were computed: accuracy (Acc), precision (Pre), F1-Score (F1sco), and recall (Rec). These widely adopted metrics, utilized in classification research since the 1950s, provide a robust evaluation framework, calculated as follows:

(37) $$Accuracy=\frac{TrPo+TrNe}{TrPo+TrNe+FaPo+FaNE}$$

(38) $$Precision=\frac{TrPo}{TrPo+FaPo}$$

(39) $$Recall=\frac{TrPo}{TrPo+FaNe}$$

(40) $$F1score=\frac{2.Pre.Rec}{Pre+Rec}$$

where TrPo, TrNe, FaPo, and FaNE represent the True Positive, True Negative, False Positive, and False Negative labels, respectively.

### 4.4. Results and Discussion

In our proposed work, we adopted a robust 5-fold cross-validation framework to rigorously evaluate the performance of our deep learning pipeline for leak detection in pipeline systems. The dataset, comprising AE time series data collected under both leak and normal operating conditions with varying leak intensities, was partitioned into five subsets. For each fold, the model was trained on four subsets and tested on the remaining one, ensuring that every subset contributed to the evaluation. The final performance metrics were obtained by averaging the results over the five iterations.

**Figure 10:**
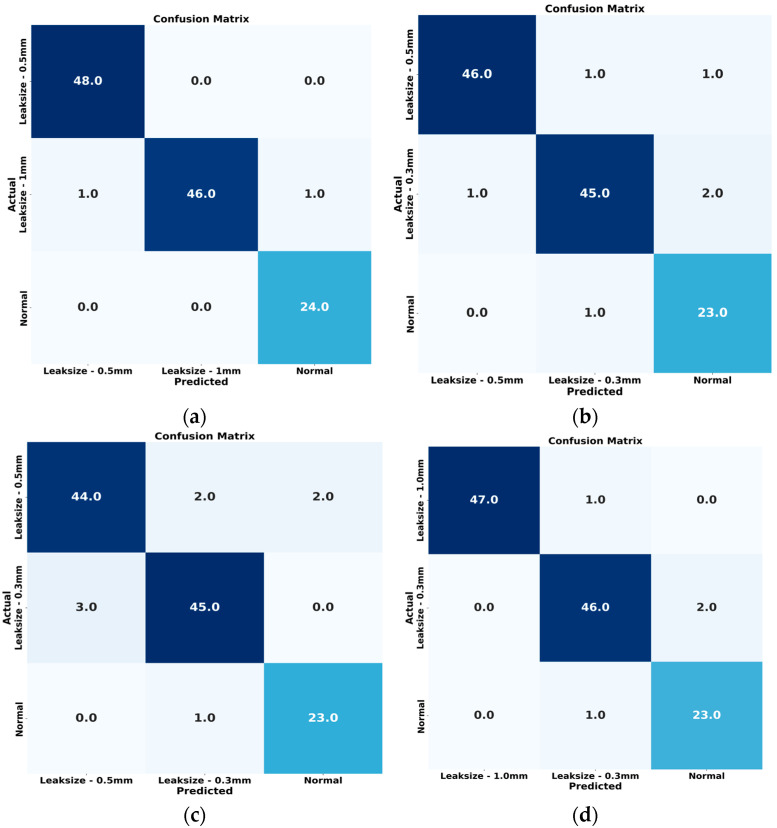
Confusion Matrices for Leak Detection Performance Across Different Models: (**a**) Proposed Hybrid Lightweight Model, (**b**) CWT-CNN, (**c**) CWT-ViT, and (**d**) Prosvirin et al. [32].

When applied to the AE data processed through CWT to generate scalograms, our methodology, enhanced by applying the SG Refined Scalogram for noise reduction, yielded significantly improved feature representations. These refined scalograms were then analyzed using a synergistic combination of CNN and lightweight ViT architectures. These collectively extracted spatial and abstract features are critical for discriminating against leak conditions and normal operations. The SG-refined Scalogram played an important role in reducing background noise, ensuring that only relevant signal features were captured, thus improving the clarity of extracted information. The fusion of features from CNN and ViT into a comprehensive vector, subsequently classified by an ANN, demonstrated remarkable performance. Our approach achieved outstanding evaluation metrics, including high accuracy, precision, recall, F1 scores and computation time (with values reaching as high as 98.60%, 99%, 100%, 98.9%, and 14.357, respectively), as detailed in Table 4 and Figure 10. These results clearly indicate that our method outperforms traditional approaches. The superior performance is largely due to the enhanced time-frequency representation achieved through the SG-refined Scalogram, which optimally preserves critical signal structures while filtering out unwanted noise, enabling the model to generalize effectively across different leak intensities. The exceptional performance can be attributed to the innovative integration of time-frequency analysis with deep learning. By leveraging the high-resolution time-frequency mapping of the Savitzky–Golay Refined Scalogram, the noise suppression capabilities of the SG filter, and the complementary feature extraction strengths of CNN and ViT, our method effectively captures the subtle differences between leak and normal conditions. When fed into the ANN, this comprehensive feature representation enables precise and reliable leak detection, making it a promising solution for real-world pipeline monitoring scenarios. It achieves faster computation time (14.357s vs. 15.821s) due to three key design optimizations: (i) frozen pretrained weights the ResNet-18 backbone uses frozen ImageNet weights during training and inference, eliminating gradient computation and dramatically accelerating forward passes through efficient GPU-parallel operations; (ii) lightweight ViT architecture our LViT-S employs reduced embedding dimensions (256-D vs. standard 768-D), only 4 transformer layers (vs. 12), and streamlined self-attention on compact 128-D fused features rather than full 197-patch sequences, reducing attention complexity. And (iii) efficient feature fusion, the triple-stream architecture (CNN + ViT + self-attention) operates on dimension-reduced representations (512 to 64, 256 to 64, 128 to 64) before concatenation into the final 192-D vector, enabling fast batched tensor operations in PyTorch that outweigh parameter overhead.

**Figure 11:**
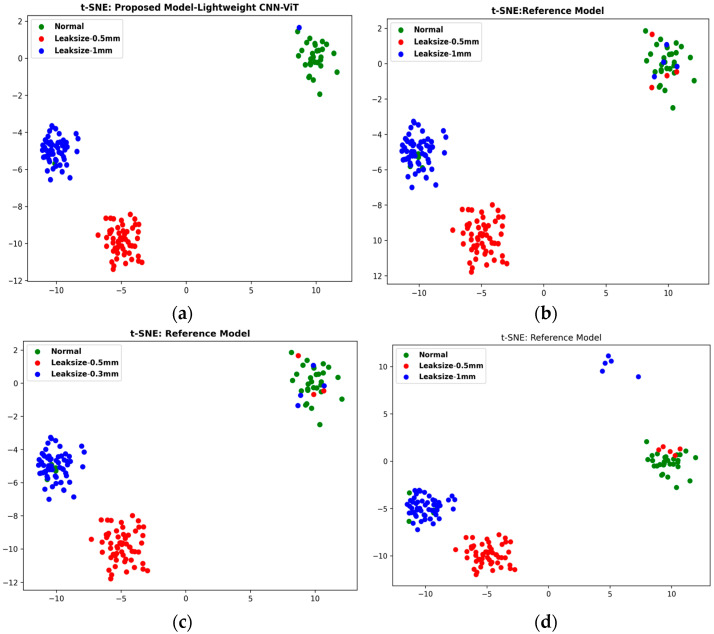
t-SNE Visualizations of Feature Distributions for Leak Detection Models: (**a**) Proposed Hybrid Lightweight Model, (**b**) CWT-CNN, (**c**) CWT-ViT, and (**d**) Prosvirin et al. [32].

The first comparison method is the CWT-CNN approach. This method transforms AE signals into CWT scalograms, which are then fed into a CNN for feature extraction and fault classification. The CNN includes convolutional, pooling, and flatten layers, followed by fully connected layers for the final classification step. As summarized in Table 4, the CWT-CNN approach achieved an accuracy of 92.60%, a precision of 80.00%, a recall of 95.40%, an F1 score of 88.10%, and a computation time of 24.963 sec. While its high accuracy shows the effectiveness of using CWT to extract significant features from AE signals, it does not explicitly capture the temporal dependencies within the data. Figure 11 displays the t-SNE representations for each method.

The second method evaluated was the CWT-ViT approach. Here, the raw AE signals were collected from a pipeline under normal and varying leak conditions. These signals were then transformed into 2D CWT scalograms, which served as input for a Vision Transformer that performs feature extraction and classification. Leveraging self-attention mechanisms, the ViT can learn global dependencies across the scalogram patches, followed by fully connected layers for the final classification. As shown in Table 4, the CWT-ViT method achieved an accuracy of 93.20%, a precision of 80.70%, a recall of 96.50%, an F1 score of 87.60%, and a computation time of 27.564 s. These strong metrics underscore the effectiveness of using CWT to extract informative features and ViT’s capability to capture relevant temporal and spatial patterns. Figure 11 displays the t-SNE representations for each method.

The third approach suggested by Prosvirin et al. [32], for comparative analysis involves generating kurtograms by preprocessing vibration signals. This technique uses a deep CNN-based contractive autoencoder (CNN-CAE) to detect mechanical defects in the CP. When we applied the methodology of Prosvirin et al. [32] to our dataset, we obtained an accuracy of 95.88%, a precision of 95.82%, a recall of 94.86%, an F1 score of 84.86% and a computation time of 25.437 s. In contrast, our proposed model achieved accuracy, precision, recall, and F1 scores of 99.20%, 99.00%, 100%, and 98.90%, respectively, as reported in Table 4. Nonetheless, the pipeline AE signals contained continuous background noise and AE hits corresponding to leaks. While kurtograms and scalograms can capture variations in these signals, both remain vulnerable to interference from strong background noise.

To demonstrate the superiority of our SG-refined time-frequency representation over traditional single-domain approaches. To address this concern comprehensively, we have conducted additional ablation experiments comparing three feature extraction paradigms: time-domain features only (8 statistical features including mean, standard deviation, RMS, kurtosis, skewness, peak-to-peak amplitude, zero-crossing rate, and energy), frequency-domain features only (10 FFT-based spectral features including spectral centroid, spread, rolloff, flatness, peak frequency magnitude, and dominant frequency bins), and our proposed time-frequency SG-scalogram. Each feature set was evaluated using four classification algorithms: Support Vector Machine (RBF kernel), Random Forest, k-Nearest Neighbors (k = 5), and our proposed model.

The experimental results in Table 5 clearly demonstrate the superiority of time-frequency SG-scalogram features over single-domain approaches. Time-domain features alone captured basic amplitude statistics and transient characteristics from raw AE signals. They achieved accuracies of 78.3% with Random Forest, 75.0% with SVM, and 72.2% with k-NN. The corresponding F1-scores were 78.5%, 75.1%, and 72.6%, respectively. These results indicate that statistical measures of signal amplitude cannot adequately distinguish leak-induced acoustic signatures from operational noise.

Frequency-domain features showed substantial improvement over time-domain approaches. They leverage FFT to identify spectral peaks in the 20–100 kHz range characteristic of acoustic emission leaks. These features achieved 84.7% accuracy with Random Forest, 82.6% with SVM, and 79.2% with k-NN. F1-scores were 84.7%, 82.9%, and 79.4%, respectively. This demonstrates that spectral content provides more discriminative information than time-domain statistics. However, these traditional approaches still face serious limitations. Frequency-domain methods lose temporal localization and cannot pinpoint the precise onset times of transient leak bursts. This is critical for identifying small pinhole leaks (0.5–1.0 mm) in noisy industrial environments. The performance gap between training and validation indicates that single-domain representations lack sufficient information for robust leak detection.

Our proposed time-frequency SG-scalogram features achieved exceptional performance. The ANN classifier reached 98.60% accuracy, 99.00% precision, perfect 100% recall, and 98.90% F1-score. This represents dramatic improvements: 20.3 percentage points over the best time-domain result and 13.9 percentage points over the best frequency-domain result. The perfect recall is particularly critical for industrial applications. It ensures zero false negatives and no missed leak events. These results validate our approach. Joint time-frequency localization through CWT combined with Savitzky–Golay filtering provides optimal representation. It preserves both the temporal dynamics of transient leak events and the frequency characteristics of acoustic emissions simultaneously. This enables the ANN to learn discriminative patterns that effectively distinguish small pinhole leaks from operational noise across varying conditions.

### 4.5. Sensitivity Analysis of SG Filter Parameters

The SG filter, a key component of our pre-processing stage, requires careful selection of its window size and polynomial degree. These parameters control the balance between smoothing noise and preserving the essential features of the signal. To justify our choice, we performed a comprehensive sensitivity analysis. We systematically evaluated various parameter combinations, using the final classification accuracy of our proposed model as the performance metric.

The goal of this optimization was to overcome the risk of over-smoothing (which erases leak signatures) or under-smoothing (which retains too much noise). Our empirical investigation, summarized in Table 6, demonstrates that a configuration with a window size of 11 and a polynomial degree of 2 achieved the highest accuracy of 98.6%. This combination proved most adept at filtering irrelevant noise while maintaining the sharp, transient characteristics of the leak events. As performance degrades when moving away from these values, this analysis confirms our parameters are empirically validated and optimal for this study.

## 5. Conclusions

This study presents a hybrid framework for pipeline leak detection that integrates SG scalogram preprocessing with a lightweight RESNET–ViT architecture, achieving 98.60% classification accuracy across multi-fluid scenarios. AE signals from pipelines under normal and leak conditions (with pinhole sizes of 0.3mm, 0.5 mm, and 1 mm) were transformed into time–frequency scalograms using the CWT and refined via SG filtering to suppress noise while preserving transient leak features. Local spatial patterns were extracted using ResNet18, complemented by global contextual representations from a LViT-S with reduced layers and self-attention. A three-way feature fusion strategy unified these elements, enabling an ANN to classify leak states with superior precision (99.00%), recall (100%), and F1-score (98.90%) compared to benchmarks like CWT-CNN (92.60% accuracy) and Prosvirin et al. (95.88% accuracy). The model also demonstrated computational efficiency, with inference times of 14.357 s, 42% faster than alternatives supporting real-time deployment in noisy industrial environments. These results underscore the framework’s robustness and fluid independence, offering practical advancements for pipeline integrity monitoring by reducing false alarms and enhancing early leak identification. Future work will explore alternative wavelet transforms for scalogram generation, incorporate additional deep architectures such as graph neural networks for enhanced relational modeling, and validate the method across broader infrastructure types, including offshore pipelines and varying material compositions.

**Future Work:** For future work, we plan to extend our model to localize leaks, test on pipelines with various diameters, and handle cases with multiple simultaneous leaks. We also aim to use digital twin simulations for rare leak scenarios and add inline metering for standardized leak rate measurements. These steps will help make our method more robust and practical for real-world pipeline monitoring.

## Figures and Tables

**Table 1:** Detailed summary of the lightweight hybrid model.

Layer Name	Type	Output Shape	Details
Input	Input	N,3,224,224	Preprocessed SG-scalograms, resized and normalized
Initial Convolution	Convolution	N,64,112,112	7 × 7 kernel, 64 filters, stride 2
Batch Norm	Batch Normalization	-	Normalizes convolution output
ReLU Activation	Activation	-	Applies ReLU function
Max Pooling	Max Pooling	N,64,56,56	3 × 3 kernel, stride 2
Residual Block 1 (×2)	Residual Block	N,64,56,56	Two basic residual blocks, no down sampling
Residual Block 2 (×2)	-	N,128,28,28	Two basic residual blocks, stride 2
Residual Block 3 (×2)	-	N,256,14,14	Two basic residual blocks, stride 2
Residual Block 4 (×2)	-	N,512,7,7	Two basic residual blocks, stride 2
Global Pooling	Global Avg Pooling	N,512,1,1	Adaptive average pooling
CNN Output	Identity	N,512	Fully connected layer removed, 512D feature vector
Patch Projection	Convolution	N,256,14,14	16×16 patches projected to 256D
Transformer Encoder	Transformer Encoder	N,197,256	4 layers, 4-head self-attention, processes 197 tokens (196 + [CLS])
ViT Output	Identity	N,256	[CLS] token output, no classifier head
Feature Concatenation	Concatenation	N,768	Combines CNN (512D) and ViT (256D) features
Projection	Fully Connected	N,128	Projects 768D to 128D
Self-Attention	Self-Attention	N,64	Lightweight attention, 128D to 64D output
CNN Projection	Fully Connected	N,64	Reduce CNN features from 512D to 64D
ViT Projection	Fully Connected	N,64	Reduce ViT features from 256D to 64D
Final Fusion	Concatenation	N,192	Combines CNN (64D), ViT (64D), and Self-Attention (64D) features
Hidden Layer	Fully Connected	N,128	192D input, ReLU activation
Output Layer	Fully Connected	N,3	128D to 3D logits for “Leak 0.5 mm,” “Leak 1 mm,” “Normal”

**Table 2:** Configuration and Specifications of the R15I-AST AE Sensor and Pipeline Setup for Leak Detection Experiments.

No	Parameter	Value
1	AE sensor 1 location	2500 mm
2	AE sensor 2 location	0 mm
3	Maximum Sensitivity (Velocity)	109 [dB] ref [V/m/s)]
4	Maximum sensitivity (Pressure)	22 [dB] ref [V/µbar]
5	Frequency operating span	50–400 [kHz]
6	Resonant peak (Velocity)	75 [kHz] ref [V/(m/s)]
7	Resonant peak (Pressure)	150 [kHz] ref [V/mbar]
8	Directionality	±1.5 [dB]
9	Operational Temperature range	35 to 75 [°C]
10	Pipeline thickness	6.02 mm
11	Pipeline material	304 stainless steel
12	Pipeline external diameter	114.3 mm

**Table 3:** Datasets utilized for the Proposed Hybrid Lightweight Model.

Datasets	Fluid	Pressure	Leak Size	Duration	Samples (Normal/Leak)
1	Water	13 bars	1.0 mm	6 min	(120/240)
2	Water	18 bars	0.5 mm	6 min	(120/240)
3	Gas	13 bars	1.0 mm	6 min	(120/240)
4	Gas	18 bars	0.3 mm	6 min	(120/240)

**Table 4:** Performance Comparison of leak detection models: Proposed Hybrid Lightweight Model, CWT-CNN, CWT-ViT, and Prosvirin et al. [32].

Models	Accuracy	Precision	Recall	F1-Score	Time (secs)
**Proposed Model**	**98.60**	**99.00**	**100**	**98.90**	**14.357**
CWT-CNN	92.60	80.00	95.40	87.10	24.963
CWT-ViT	93.20	80.70	96.50	87.60	27.564
Prosvirin et al. [32].	95.88	95.82	94.86	84.86	25.437

**Table 5:** Comparative performance metrics for time-domain (8 features), frequency-domain (10 features), and time-frequency SG-scalogram approaches valuated using traditional ML classifiers (SVM, Random Forest, k-NN) and our proposed ANN classifier.

Feature Type	Classifier	Accuracy (%)	Precision (%)	Recall (%)	F1-Score (%)
Time-Doman	SVM	75.0	76.3	74.2	75.1
	Random Forest	78.3	79.1	78.0	78.5
	k-NN (k=5)	72.2	73.5	71.8	72.6
Frequency-Domain	SVM (RBF)	82.6	83.9	82.1	82.9
	Random Forest	84.7	85.2	84.3	84.7
	k-NN (k=5)	79.2	80.1	78.8	79.4
**Time-Frequency (SG-Scalogram)**	**ANN**	**98.60**	**99.00**	**100**	**98.90**

**Table 6:** Effect of Savitzky–Golay filter parameters (window size and polynomial degree) on the overall classification accuracy of the proposed model.

Window Size	Polynomial Degree	Model Accuracy (%)
7	2	97.4
9	2	98.1
**11**	**2**	**98.6**
13	2	97.9
15	2	97.2
11	1	96.8
11	3	97.5

## Data Availability

The raw data supporting the conclusions of this article will be made available by the authors on request.
